# Development of a personalized decision aid for breast cancer risk reduction and management

**DOI:** 10.1186/1472-6947-14-4

**Published:** 2014-01-14

**Authors:** Elissa M Ozanne, Rebecca Howe, Zehra Omer, Laura J Esserman

**Affiliations:** 1 Department of Surgery, Institute for Health Policy Studies, University of California at San Francisco, San Francisco, CA, USA; 2 The Dartmouth Institute for Health Policy and Clinical Practice, Geisel School of Medicine at Dartmouth, 35 Centerra Parkway, Lebanon, NH 03766, USA; 3 Department of Surgery, University of California at San Francisco, San Francisco, CA, USA; 4 University of Massachusetts, Worcester, USA

**Keywords:** Breast cancer, Decision aid, Risk assessment, Risk reduction, Decision making, Primary care

## Abstract

**Background:**

Breast cancer risk reduction has the potential to decrease the incidence of the disease, yet remains underused. We report on the development a web-based tool that provides automated risk assessment and personalized decision support designed for collaborative use between patients and clinicians.

**Methods:**

Under Institutional Review Board approval, we evaluated the decision tool through a patient focus group, usability testing, and provider interviews (including breast specialists, primary care physicians, genetic counselors). This included demonstrations and data collection at two scientific conferences (2009 International Shared Decision Making Conference, 2009 San Antonio Breast Cancer Symposium).

**Results:**

Overall, the evaluations were favorable. The patient focus group evaluations and usability testing (N = 34) provided qualitative feedback about format and design; 88% of these participants found the tool useful and 94% found it easy to use. 91% of the providers (N = 23) indicated that they would use the tool in their clinical setting.

**Conclusion:**

BreastHealthDecisions.org represents a new approach to breast cancer prevention care and a framework for high quality preventive healthcare. The ability to integrate risk assessment and decision support in real time will allow for informed, value-driven, and patient-centered breast cancer prevention decisions. The tool is being further evaluated in the clinical setting.

## Background

Involving women in decisions related to their own health is integral to the adoption of preventative health measures. This is particularly true in efforts intended to reduce breast cancer incidence. For example, although more than 2.4 million women in the United States are projected to benefit from breast cancer risk-reducing medications such as tamoxifen or raloxifene [1,2], fewer than 1% of eligible patients elect to take them [3,4]. This low utilization rate is largely due to clinician difficulties in assessing patients’ risk, patients’ misperceptions of their own risk of developing breast cancer, and the lack of familiarity on the part of both patients and clinicians with the risks and benefits of these medications [5]. Given the prevalence of breast cancer and the efficacy of available preventive interventions [6], addressing the challenge of risk assessment and risk reduction should be a fundamental part of women’s clinical care.

Making well-informed decisions about breast cancer risk reduction requires women to weigh the benefits and risks of the options and determine how they value the possible outcomes of each. In addition, women’s risk of breast cancer and preferences for interventions around breast cancer screening and risk reduction vary widely and must be included in their decision making. In this setting, there is a need for: 1) personalized risk assessment, 2) unbiased, comprehensive education about the options for risk reduction, and 3) an opportunity for meaningful consideration of the risks and benefits involved on the part of the patient.

Decision aids are tools designed to support informed medical decision making and can facilitate efficient communication between patients and clinicians about these risks and benefits. These tools offer enhanced knowledge about interventions, allowing women the opportunity to weigh the risks and benefits of their choices. They have been shown to help patients increase their knowledge of their options, to reduce their decisional conflict, and to make decisions that align with their goals and values [7,8]. Clinical decision aids have been developed for a broad range of health issues; however, few decision aids have been developed for breast cancer risk reduction. Those that have been developed for clinical use are in the form of generic DVDs, CD-ROMs and written educational materials [9-11] designed for a single risk-reduction option or intended for small subgroups of women, such as women considering genetic testing or women with a known genetic mutation [3,12-16].

While these tools have been shown to be both feasible and beneficial, they have a number of limitations. Most are not tailored to patients’ individual breast cancer risk or health characteristics. Also, they are not designed for use within the primary care setting, where risk reduction efforts are likely to have the strongest impact [7]. Furthermore, informed decision-making in the clinical setting can be impeded by significant demands on physicians’ time, incomplete and often conflicting data, and rapidly changing scientific evidence and consensus recommendations. These factors often hinder informed decision-making in the clinical setting, underscoring the value of decision support tools in this environment [17-19].

To address these decision-making challenges to breast cancer risk reduction, we developed a comprehensive framework to guide and inform consultations [20]. Based on this framework, we developed a decision aid prototype, intended to be used with patients being seen in a high-risk clinic. It encourages informed decision making and aims to align patient decisions with patient values and current clinical knowledge. The tool was designed for collaborative use between patients and clinicians, including primary care physicians, cancer risk clinicians, and genetic counselors, to provide both personalized risk assessment and decision support pertaining to breast cancer risk reduction. The design was intended to be flexible so that the tool could be used at any point in a clinical visit, including before the visit as an educational primer.

The design of the decision aid prototype and assessment of its clinical feasibility and effectiveness has been explained in depth previously [21]. It was developed and implemented in the breast cancer prevention program at UCSF, and piloted in a feasibility trial comparing consultations performed using it to standard consultations. It was found to be clinically feasible and acceptable to both patients and providers in this setting. Although not powered to detect an impact in efficacy, this initial evaluation appeared to show beneficial effects in terms of patient knowledge and satisfaction.

To extend the generalizability and broaden the applicability of the decision aid, we transitioned the prototype into a web-based tool called BreastHealthDecisions.org designed for multiple clinical settings, including primary care clinics. In this paper, we describe the process used to develop BreastHealthDecisions.org, the results of the usability testing, and how stakeholder input was incorporated into the generalizable web-based tool.

## Methods

BreastHealthDecisions.org was developed through an iterative research process that allowed for continuous improvement of the decision support tool. Research involved assembling a team of expert advisors, developing the decision aid prototype, and evaluative work with key stakeholders.

### Theoretical framework

Our earlier framework and decision aid were guided by the decision analytic framework developed by Howard and colleagues [22,23]. The theoretical basis of decision analysis consists of the following elements: 1) appropriate framing, 2) creative alternatives, 3) quality information, 4) consistent preferences, 5) clear logic, and 6) commitment to action on the part of the decision maker. According to the Howard Framework, attention to these six elements is required for making quality decisions and were incorporated into our decision aid.

### Development process

The content, format, and style of the decision aid were developed using current literature on risk communication [24-28] as well as the advice of clinicians, health policy analysts, decision analysts, information architecture experts, and an advisory board. A scientific advisory board of experts in breast cancer risk, and risk reduction and management, was assembled to guide the decision tool development process. The advisory board consisted of leading experts in the field of breast cancer prevention, including surgeons, oncologists, genetic counselors, decision scientists, statisticians, primary care physicians, epidemiologists, behavioral scientists, and patient advocates. The advisory board met as a group to review the first version of the decision aid and was consulted on a regular basis regarding changes and improvements in the decision aid.

The web tool, developed by Drs. Ozanne and Esserman and colleagues at UCSF, generates tailored patient-specific risk assessments using established risk models (including Gail, BRCApro, Claus, BCSC density) based on patient data [29-32]. Consultants from MAYA Viz, a company focused on web design, provided format and style guidance and aided the development of the tool’s information architecture [33]. The International Patient Decision Aid Standards (IPDAS) were also used to guide the development of the decision aid where appropriate [34]. The IPDAS were designed primarily for patient facing decision aids, while our tool is intended for use by a patient and clinician dyad. Therefore we consulted the relevant criteria for our tool.

We have obtained the domain name http://www.BreastHealthDecisions.org to support open access for the decision aid. The web-based version is designed for use by both patients and their providers, specifically breast specialists, gynecologists, and primary care physicians. The tool uses patent risk factors including family history of cancer and patient biomarkers–such as atypia, breast density, and genetic mutations–to generate estimates of risk and intervention benefits.

The web-based tool was designed with the following core characteristics:

• *individual tailoring* to patients’ breast cancer risk and overall health using validated risk assessment models,

• applicability to the *primary care setting*, by providing content relevant to the general population of women,

• addresses the *risk of side effects* from risk reduction measures, one of the main barriers to use,

• designed for use *at the time of the consultation*, facilitating communication between patients and clinicians, and

• *web-based platform*, providing an easily accessible platform for both patients and clinicians that can be updated rapidly and disseminated broadly.

### Usability testing

To evaluate the content and usability of BreastHealthDecisions.org, qualitative data was collected through usability testing with patients and health care providers. Discussions with the primary users of the decision aid and relevant experts were conducted to provide us with information about how to optimize the decision aid for future use in clinical settings. We aimed to elicit feedback on the content, structure, ease of use, and design of the decision aid.

### Participants

We targeted three groups of participants: patients, providers, and decision scientists and experts in shared decision-making. The participants provided verbal consent for the focus group and semi-structured interviews, according to the research protocol that was reviewed and approved by the institutional review boards at the University of California, San Francisco and Massachusetts General Hospital.

The patient focus group consisted of breast cancer survivors who were recruited from a list of volunteers at a large cancer center. We chose breast cancer survivors to avoid raising patient anxiety in women who had not previously been diagnosed with breast cancer. We also chose them because they are knowledgeable about the issues surrounding breast cancer and could provide their opinion about the decision aid, but were not currently facing the specific decisions described in the decision aid. The health care provider participants were recruited at the 2009 San Antonio Breast Cancer Symposium (SABCS), held in San Antonio, TX. These participants, targeted as potential users of the decision aid, were asked to complete pilot testing at SABCS. Targeted evaluators included genetic counselors, primary care physicians, surgeons, oncologists, and nurse practitioners.

The final group of participants consisted of experts in the field of decision sciences and shared decision-making. These participants were recruited from the 2009 International Shared Decision Making Conference (ISDM), held in Boston, MA.

### Data collection

A case study for a high-risk woman was presented to the patient focus group participants and the group walked through the decision aid as it would be used in a consultation. After the presentation, a group discussion was initiated by an experienced facilitator using a semi-structured guide designed by the research team. The discussion lasted for approximately two hours and was transcribed. The participants commented on the usefulness of each section of the decision aid. Participants also gave advice about the relevance of the content, the ease of use of the design, and general comments about the structure of the tool.

The health care provider participants viewed the web-based decision aid and completed one-on-one interviews with the principal investigator of the study. The interviews used a semi-structured interview guide that asked the participants about the appropriateness and usability of the decision aid in different clinical settings. The decision scientist experts went through a similar process, and were asked questions relating to the design and structure of the decision aid with respect to patient decision-making. All participants in the interview groups were also asked about the scientific relevance and validity of the content used in the decision aid and the usability of the decision aid. These participants were also invited to submit general comments about the structure and design of the tool.

Each participant across the three groups was asked to indicate: 1) if they found the decision aid useful and 2) if they found the decision aid easy to use. Clinicians were asked additionally: 3) if they would use the tool in their clinics.

## Results

### Usability testing

Information gathered from the focus group evaluations and usability testing provided qualitative feedback about format and design of the decision aid. Six breast cancer survivors participated in the patient focus group recruited from a patient research registry. Twenty-three participants completed pilot testing of the decision aid at SABCS, and five participants completed usability testing of the decision aid at ISDM. Table 1 presents relevant characteristics of these participants.

**Table 1:** Summary of participant characteristics

	**Patients**	**SABCS participants**	**ISDM participants**
Total	6	23	5
Age	44–67	*	*
Gender			
Female	6	17	3
Male	0	6	2
Characteristics	Breast cancer survivors:	Specialties:	Decision science experts
	All at least one year out from initial diagnosis	Surgeons	
		Medical oncologists	
		Radiologists	
		Genetic counselors	
		Psychologists	
		Nurse practitioners	
Demographics			
Caucasian	6	*	*

*Not reported.

Overall, the participants found the usability of the decision aid to be favorable. As shown in Table 2, 91% of the 23 clinicians interviewed said they would use the decision aid in their clinic. Most of the patients and providers who were interviewed found the decision aid useful and easy to use. Comments of the participants provided in the focus group are presented in Table 3. These comments are representative of the overall opinion, and form the backbone of the consensus arrived at during the focus group.

**Table 2:** Usability results from testing groups

	**Found the decision aid useful**	**Found the decision aid easy to use**	**Clinicians would use decision aid in their clinic**
	**N (%)**	**N (%)**	**N (%)**
Patient group (N = 6)	5 (83)	6 (100)	NA
Providers at SABCS (N = 23)	21 (91)	22 (96)	21 (91)
Experts at ISDM (N = 5)	4 (80)	4 (80)	NA
**Total (N = 34)**	**30/34 (88)**	**32/34 (94)**	**21/23 (91)**

**Table 3:** Focus group consensus comments

***Topic***	***Statement representative of group consensus***
*General reception:*	*“Glad to hear there are prevention tools and methods”*
*Usability:*	*“The tool is easy to read and follow”*
*Personalization:*	*“I want to see risks for my situation”*
*Design:*	*“The color and design are nice”*
*Format:*	*“I want all the information to be accessible online”*

### Finalized content (by Modules)

Based on the feedback of the pilot testing (previously reported) [21] and the usability testing reported here, the following format for the decision aid was decided upon. A modular design was used in the development of this tool to allow each patient to focus on the relevant information to her situation. The participants indicated that this kind of design was important to allow patients and providers to view only the content relevant to them and to easily skip from one section to another. Providers also felt that the ability to view sections independently is intended for use within the clinical visit.

The summary results indicate that the decision aid was designed in a way that was easy to follow and easy to use. The predominant opinion of the participants was that it was important for the tool to remain online to allow patients to refer to it after the consultation. It was also important to the participants for the decision aid to be tailored to each patient’s individual risk of developing breast cancer.

**Figure 1:**
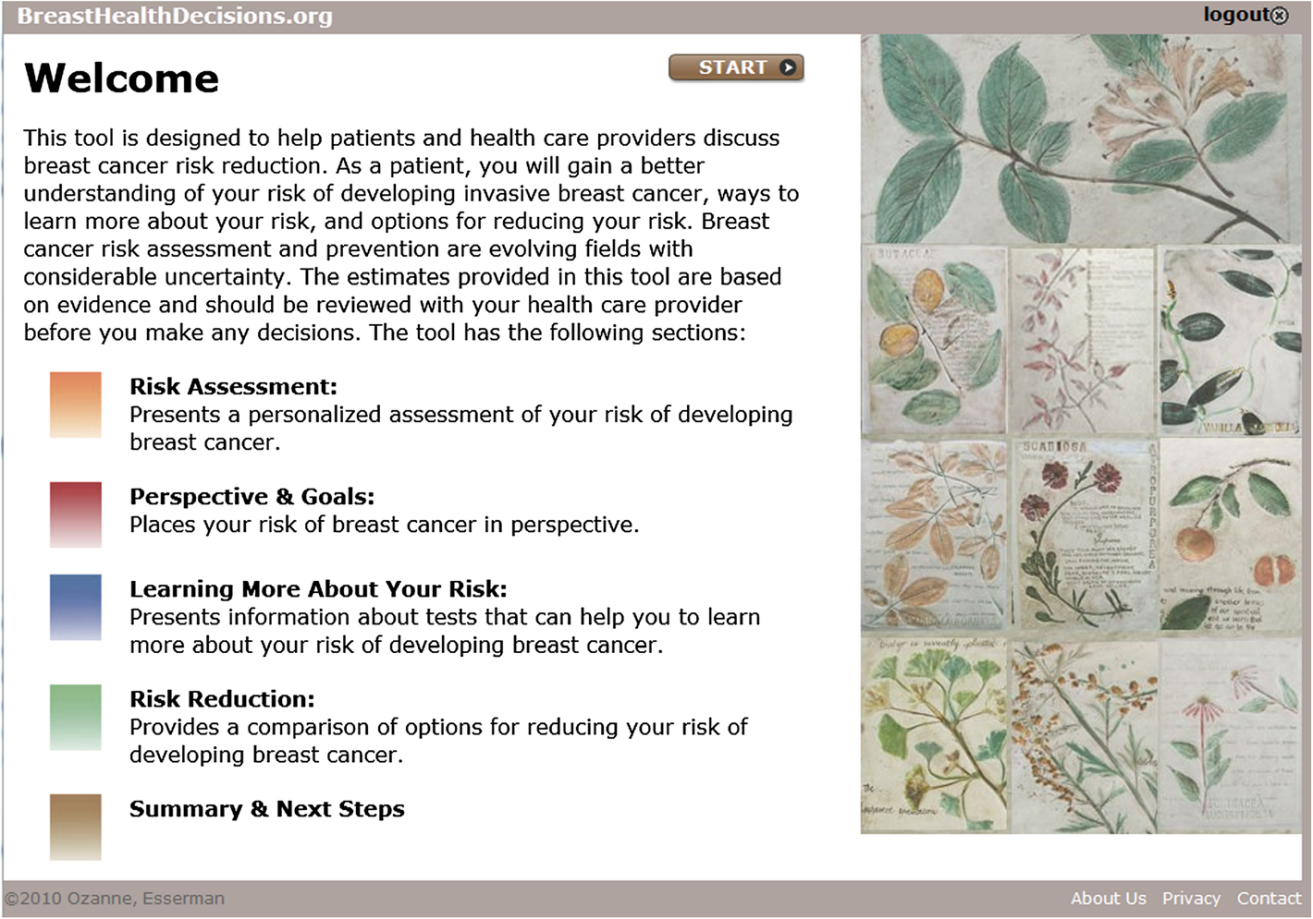
Overview of BreastHealthDecisions.org.

Based on feedback from the advisory board and scientific advisors, the content was divided into five cohesive sections presented below. The design of the tool will allow users to skip modules that are less relevant and spend more time on modules of greater interest. Figure 1 presents an overview of the decision content.

1) Risk Assessment: Presents a personalized assessment of a woman’s risk of developing breast cancer. Five-year and lifetime risk scores are calculated using four validated models (Gail, Claus, BRCApro, BCSC Density) [29-32] to provide a more complete initial screen identifying both family history of breast cancer and biological or hormonal exposure risk factors. The decision aid was designed to that additional models can added in the future.

2) Perspective & Goals: Compares an individual woman’s risk of breast cancer to other women and to risk of other causes of death. Individual breast cancer risks are compared to an average, age-matched woman to offer better risk discrimination and to improve risk communication. Individual breast cancer risks are also displayed in the context of data on leading causes of death, such as heart disease and lung cancer, to help prioritize breast cancer risk.

3) Learning More About Your Risk: Presents information about tests that can help a woman learn more about her risk of developing breast cancer. Biomarkers (such as atypia, breast density and BRCA1 or BRCA2 mutations) stratify patients by risk and identify the likely benefit from an intervention.

4) Risk Reduction: Provides a comparison of options for reducing a woman’s risk of developing breast cancer. Presents information about lifestyle changes, chemoprevention, and surgical options while reflecting quality of life trade-offs.

5) Summary & Next Steps: Captures the synopses and decisions related to risk-assessment and risk-reduction options for patients and can be incorporated into medical records.

## Discussion

In this paper we have described how BreastHealthDecisions.org has been developed and evaluated as a personalized decision aid that can be used in a primary care setting to facilitate breast cancer risk reduction discussions. In developing the tool, we have used an iterative research process that incorporated feedback from a team of expert advisors, focus group data and usability interviews with key stakeholders and potential users of the decision aid. This process is aligned closely with that developed by Elwyn and colleagues that has been published since the collection of these data [35]. The resulting tool is tailored to individual women and their risk levels and allows for flexible use during a clinical visit. BreastHealthDecisions.org is a comprehensive, personalized decision aid for breast cancer risk reduction for use in the primary care setting. It fills the need for an efficient and effective tool that can facilitate the tasks of risk assessment and risk communication about breast cancer risk-reduction options.

The efficacy of strategies to reduce the risk of developing breast cancer has been proven, but these interventions are not being implemented. In contrast, although the value of screening mammography is intensely debated, participation rates for screening are between 72 and 80%. Incorporating risk stratification and risk-reduction into routine clinical consultations would provide an opportunity to engage women in the larger context of their overall breast health, and could identify highest risk women who would derive the greatest benefit from these interventions.

Studies of women’s decisions about breast cancer prevention and screening conclude that women have a tendency to overestimate both their risk of breast cancer and the risks of side effects associated with risk-reducing interventions, leading to suboptimal decision making. The majority of a cohort of 75 women who had undergone prophylactic bilateral mastectomy had a significantly exaggerated perception of their risk at the time they made their mastectomy decisions [36]. These women may have chosen this aggressive intervention due to their inaccurate understanding of breast cancer risks, highlighting the need for tools such as BreastHealthDecisions.org to help providers and patients achieve a more informed view about the risks an individual faces and the impact of risk reducing options.

Additionally, women often focus on breast cancer risk, even though they may be at greater risk for other diseases such as heart disease or lung cancer, risks that can be reduced substantially through lifestyle changes. BreastHealthDecisions.org was designed to put an individual’s risk of breast cancer in context with the other health threats she may face in an effort to combat this bias and help women make more informed decisions about their health.

### Strengths and limitations of development

Although we aimed to design our tool with the greatest possible generalizability, the web-based nature of BreastHealthDecisions.org necessitates the use of the internet and therefore will not be accessible to women or clinical settings without readily accessible computers. Despite this, it has been designed to be applicable to all women, regardless of their risk level, which will broaden its generalizability. The research team is currently working on corresponding paper-based tools for environments where use of the internet is limited. Additionally, the decision aid would be most effective if it were able to develop a personalized risk of breast cancer in addition to comparing individualized competing health risks. However, there are currently no risk assessment models that perform this type of comprehensive risk prediction.

Variability among providers in communication skills and knowledge about breast cancer risk reduction may impact the effectiveness of the tool. For example, a provider may find it more important to spend time discussing the risk of heart disease with a patient, even if the patient herself is more concerned about breast cancer risk. Alternatively, the decision tool could prompt the provider to focus on breast cancer risk reduction even though the patient is first inclined to discuss other health issues. The tool is designed to account for this discrepancy in concern by presenting breast cancer risk information in the context of other risks, such as heart disease or diabetes.

### Strengths and limitations of evaluation

Evaluating BreastHealthDecisions.org through a focus group enabled us to collect a range of opinions about the usability and appropriateness of the tool. Using breast cancer survivors for our patient focus group meant that they were able to reflect on their personal experiences and concerns to provide feedback about the content and delivery of the tool, while their status as survivors made them less likely to react anxiously to the information. However, it is possible that survivors may have had a different set of issues than women at risk of developing breast cancer. Recruiting at scientific conferences resulted in a diverse set of clinicians and allowed for multiple testing environments.

In both the pilot study and the focus group, we were unable to enroll participants from diverse socioeconomic backgrounds. The large majority of participants were affluent Caucasian women with post-secondary education. It is a goal for future evaluation to analyze the feasibility and effectiveness of using BreastHealthDecisions.org with a more diverse range of women.

### Future plans

We are currently implementing the decision aid within the Athena Breast Health Network, a University of California-wide consortium. The decision aid will be used with women identified to be at elevated risk. In this implementation, we will measure indicators of informed decision-making and resource utilization.

## Conclusion

BreastHealthDecisions.org represents a new approach to integrate breast cancer risk assessment and reduction into routine care and provides a framework for high quality preventive healthcare. The ability to integrate risk assessment and decision support in real time allows for informed, value-driven, and patient-centered breast cancer risk reduction decisions. The expected outcome of our decision aid is improved patient decision-making regarding breast health: women will be more informed about their personal risks and the benefits that interventions hold for them, and will make decisions aligned with their preferences. BreastHealthDecisions.org is designed to encourage appropriate use of risk-reducing interventions by identifying women at highest risk, informing patients and supporting their preferences. Ultimately, improved decisions and appropriate use of risk-reducing interventions are expected to decrease the incidence and burden of breast cancer.

## Competing interests

The authors declare that they have no competing interests

## Authors’ contributions

EO conceived of the study, participated in its design, data collection, and drafted the manuscript. RH participated in the data analysis and helped to draft the manuscript. ZO participated in the data collection, and helped to draft the manuscript. LE participated in the design of the study. All authors read and approved the final manuscript.

## Pre-publication history

The pre-publication history for this paper can be accessed here:

http://www.biomedcentral.com/1472-6947/14/4/prepub
